# Communicative competence of generative artificial intelligence in responding to patient queries about colorectal cancer surgery

**DOI:** 10.1007/s00384-024-04670-3

**Published:** 2024-06-20

**Authors:** Min Hyeong Jo, Min-Jun Kim, Heung-Kwon Oh, Mi Jeong Choi, Hye-Rim Shin, Tae-Gyun Lee, Hong-min Ahn, Duck-Woo Kim, Sung-Bum Kang

**Affiliations:** 1 Department of Surgery, Seoul National University Bundang Hospital, 300 Gumi-dong Bundang-gu, Seongnam-si, Gyeonggi-do 13620 South Korea; 2 Department of Surgery, Seoul National University College of Medicine, Seoul, South Korea

**Keywords:** Colorectal neoplasms, Artificial intelligence, Patient education as topic, Communication

## Abstract

**Purpose:**

To examine the ability of generative artificial intelligence (GAI) to answer patients’ questions regarding colorectal cancer (CRC).

**Methods:**

Ten clinically relevant questions about CRC were selected from top-rated hospitals’ websites and patient surveys and presented to three GAI tools (Chatbot Generative Pre-Trained Transformer [GPT-4], Google Bard, and CLOVA X). Their responses were compared with answers from the CRC information book. Response evaluation was performed by two groups, each consisting of five healthcare professionals (HCP) and patients. Each question was scored on a 1–5 Likert scale based on four evaluation criteria (maximum score, 20 points/question).

**Results:**

In an analysis including only HCPs, the information book scored 11.8 ± 1.2, GPT-4 scored 13.5 ± 1.1, Google Bard scored 11.5 ± 0.7, and CLOVA X scored 12.2 ± 1.4 (*P* = 0.001). The score of GPT-4 was significantly higher than those of the information book (*P* = 0.020) and Google Bard (*P* = 0.001). In an analysis including only patients, the information book scored 14.1 ± 1.4, GPT-4 scored 15.2 ± 1.8, Google Bard scored 15.5 ± 1.8, and CLOVA X scored 14.4 ± 1.8, without significant differences (*P* = 0.234). When both groups of evaluators were included, the information book scored 13.0 ± 0.9, GPT-4 scored 14.4 ± 1.2, Google Bard scored 13.5 ± 1.0, and CLOVA X scored 13.3 ± 1.5 (*P* = 0.070).

**Conclusion:**

The three GAIs demonstrated similar or better communicative competence than the information book regarding questions related to CRC surgery in Korean. If high-quality medical information provided by GAI is supervised properly by HCPs and published as an information book, it could be helpful for patients to obtain accurate information and make informed decisions.

## Introduction

The advent of the digital era of healthcare has reduced the information disparity between healthcare professionals (HCPs) and patients, which was a substantial challenge in the previous era of healthcare [1]. During the period of prevalent medical paternalism, patients could only obtain information through medical professionals [2]. However, although the patient-doctor relationship used to be more hierarchical in the past, it has recently shifted to become more horizontal, where the patient’s opinion is respected, information is explained, and patients are given the opportunity to make choices [3]. In addition, significant advances in the field of internet and other technologies have greatly improved patient access to medical information. This has alleviated the tendency of patients to rely on doctors in the medical field for clinical information about individual conditions [4].

Artificial intelligence (AI), particularly in its autonomous and interactive form, has further catalyzed the shift toward a person-centered rather than a doctor-patient relationship, emphasizing patient autonomy [5]. Generative AI (GAI), represented by the Chatbot Generative Pre-Trained Transformer (ChatGPT), has recently gained increased attention. ChatGPT, a GAI tool developed by OpenAI (San Francisco, CA), uses its learning algorithms to generate text that closely mimics human dialogue [6]. With the public unveiling of ChatGPT, several studies on the applicability of GAI in various fields have been conducted.

Many efforts have been made to apply ChatGPT to medicine, with tangible outcomes [7]. However, most studies have focused on HCPs, with less emphasis on actual patients’ perspectives. Furthermore, studies evaluating the applicability of GAI in the field of colorectal cancer (CRC) are lacking. Therefore, this study aimed to analyze responses to questions about CRC obtained from three GAI tools (GPT-4, Google Bard, and CLOVA X) in Korean and responses from a CRC information book. Furthermore, we also aimed to discuss the clinical efficacy and feasibility of GAI in the decision-making process of patients undergoing CRC surgery by comparing the responses of HCPs and patients.

## Materials and methods

### Collecting questions

This study used a dual approach to collect questions about CRC. To obtain common questions about CRC surgery from the general population, we examined the websites of hospitals rated as “1^st^ grade” in the 7^th^ National Quality Assessment of Colorectal Cancer Practice and Treatment in South Korea by the Health Insurance Review & Assessment Service of Korea (HIRA) [8]. We investigated common questions related to CRC provided on hospital websites to understand patients’ concerns. To reflect patients’ perspective, we surveyed five patients with CRC admitted to the Seoul National University Bundang Hospital and asked them to list their preoperative and postoperative concerns. From these, we selected questions for which answers could be found in the CRC information book.

### Answer derivation

A total of 10 questions were selected, four of which were obtained from the hospital websites and six from a direct patient survey. The responses to these questions were elicited from the “Information Book on Colorectal Cancer” and three GAI tools (GPT-4, Google Bard, and CLOVA X). Previous studies have evaluated the ability of GPT-4 and Google Bard to answer medical questions [9–12]. Recently, Naver Corporation (Seongnam-si, Gyeonggi-do, Republic of Korea), a global information communication technology company, developed a GAI tool called CLOVA X on August 24, 2023. CLOVA X has strengths in questions and answers (Q&A) in Korean due to its development being based in Korea. The book *100 Questions and Answers about Colorectal Cancer* (Revised Edition), which contains answers to common questions about CRC, was used as the information book. This book was written by CRC specialists following the latest treatment guidelines and textbooks published by the National Cancer Center (Goyang, Republic of Korea) on December 18, 2020 [13]. The GAI responses were obtained by inputting the questions in Korean, and each platform was prompted to generate responses in the same language. This process was conducted simultaneously at 8:00 PM KST on November 23, 2023. This study used only initial responses to prompts and questions.

### Prompt engineering

The prompts were written in English and then translated into Korean for input. The prompts used for the GAI input were as follows: (1) target your response to the patient with CRC before or after colorectal surgery; (2) minimize the use of medical jargon and write the answers so that the general public could understand them; (3) limit the length of the answer to “X” words in Korean; (4) write your answers from the perspective of the HCP; and (5) provide your responses based on recent reliable research trends related to colorectal surgery.

The GAI tool was asked to adhere to the five aforementioned rules when responding to the questions. For rule (1), we determined whether each question was related before or after surgery. For rule (3), the word count in the answer to each question in the CRC information book was used as a reference, and we aimed for a uniform response length by setting a similar word count limit for the GAI tool.

### Response evaluation

The responses were evaluated by a multidisciplinary team (MDT) and a group of patients. The MDT consisted of five members: two board-certified colorectal surgeons, one medical oncologist, one gastroenterologist, and one specialized physician-assistant nurse. For patient evaluators, the same five patients who had participated in an earlier survey on preoperative and postoperative inquiries were involved. On the basis of the review of previous studies evaluating the response abilities of GAI tools, this study used the following five evaluation criteria, specifically selecting those deemed most clinically important: accuracy of information (the medical appropriateness of the information contained in the response) [9], completeness of answer (the organic composition of the sentences and the grammatical appropriateness of the answer) [14], level of empathy (quantity and quality of empathetic expressions in the response) [7], humanness of the answer (similarity to linguistic expressions used in actual human responses) [15], and patient satisfaction (understanding of the intent of the question and consideration of the patient’s perspective) [16]. Considering the evaluators’ characteristics, the MDT evaluated the accuracy of information, and the patients evaluated patient satisfaction. Consequently, the MDT evaluated four criteria: accuracy of information, completeness of answer, level of empathy, and humanness of answer. The patient group evaluated four criteria: completeness of answer, level of empathy, humanness of answer, and patient satisfaction.

For each question, the order of the four different responses from the three GAI tools and the information book was randomized to avoid identifying the responding entity. The evaluators scored each answer on a 5-point Likert scale according to the four evaluation criteria, with a maximum score of 20 points per answer.

### Statistical analysis

Analysis of variance (ANOVA) was used to compare the mean scores between the evaluated entities, comprising the three GAI tools and the information book. This comparison was based on assessments from the MDT and patient cohorts. The homogeneity of variance, a prerequisite for ANOVA, was ascertained using Levene’s test. In instances where homogeneity was not observed, Welch’s *t*-test was used as a robust alternative. Post hoc analyses, following significant findings in ANOVA, were used to elucidate specific differences between the groups. Tukey’s test was applied in cases adhering to the homogeneity of variances, whereas Games–Howell’s test was used with Welch’s *t*-test for datasets not meeting this criterion. All statistical calculations were performed using the Social Sciences Statistical Package (version 27.0; IBM Corp., Armonk, NY, USA), with a predetermined significance level set at *P* < 0.05.

## Results

**Fig. 1:**
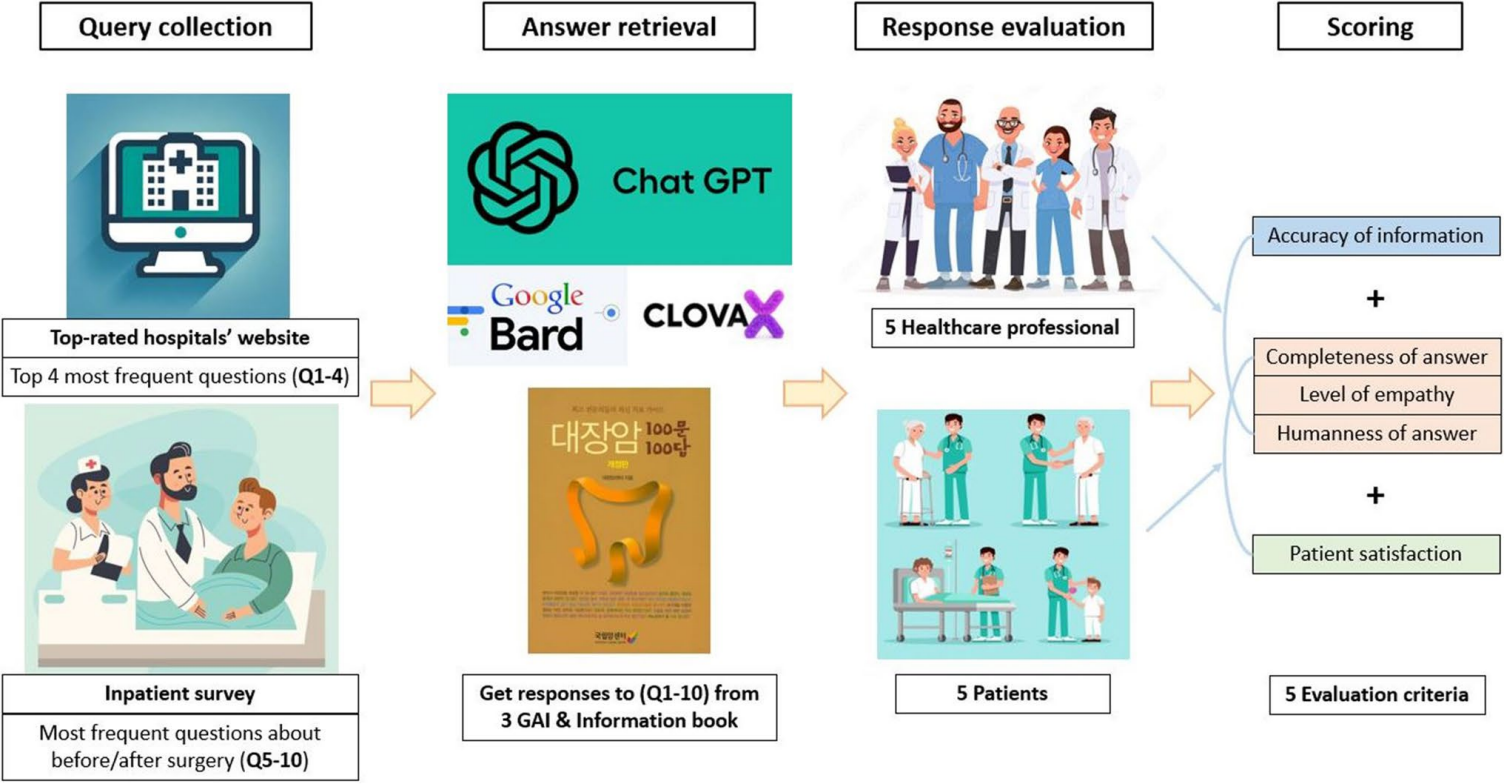
Study flowchart

The flowchart of the study is shown in Fig. 1.

### Questions from top-rated hospital websites

A search for hospital evaluations on the HIRA website revealed that 103 (46.4%) of the 222 hospitals evaluated achieved “1^st^ grade.” Of these, 64 hospitals (62.1%) featured Q&A sections for patients with CRC on their websites. All questions registered on the websites were organized, and the following four most frequently asked questions were selected: “What are the symptoms of CRC?” (57.8%), “How is CRC diagnosed?” (57.8%), “How can CRC be prevented?” (53.1%), and “What are the causes of CRC?” (40.6%) (Table 1, questions 1–4).

**Table 1:** List of 10 questions regarding colorectal cancer selected for analysis

Question number	Question	Timing of the question (preoperative/postoperative)	Word count limits (used for prompt engineering)
Q1.	What are the symptoms of colorectal cancer?	Preoperative	75
Q2.	What are the causes of colorectal cancer?	Preoperative	300
Q3.	How is colorectal cancer diagnosed?	Preoperative	150
Q4.	How can colorectal cancer be prevented?	Preoperative	125
Q5.	I have heard that the surgical approach varies depending on the location of the cancer. What are these different methods?	Preoperative	260
Q6.	How is pain managed after surgery?	Postoperative	125
Q7.	What are the dietary considerations when a colostomy is performed?	Postoperative	240
Q8.	I have been told that I have hereditary colorectal cancer; what is the likelihood of my children developing cancer?	Postoperative	120
Q9.	What are the criteria for undergoing adjuvant chemotherapy after surgery?	Postoperative	70
Q10.	What are the patterns of recurrence in colorectal cancer, and what are the treatments for it?	Postoperative	260

### Questions from the patient survey

Following a survey of five patients admitted to the hospital who addressed their inquiries before and after surgery, 37 questions were collected. Of the questions collected, 18 were related to preoperative concerns and 19 addressed postoperative inquiries. From these, we selected six commonly asked questions for which answers could be found in the CRC information book. The questions were classified as preoperative or postoperative (Table 1, questions 5–10). In total, 10 questions were selected for analysis: four extracted from the website and six obtained through the patient survey.

### Overall score assessment

**Fig. 2:**
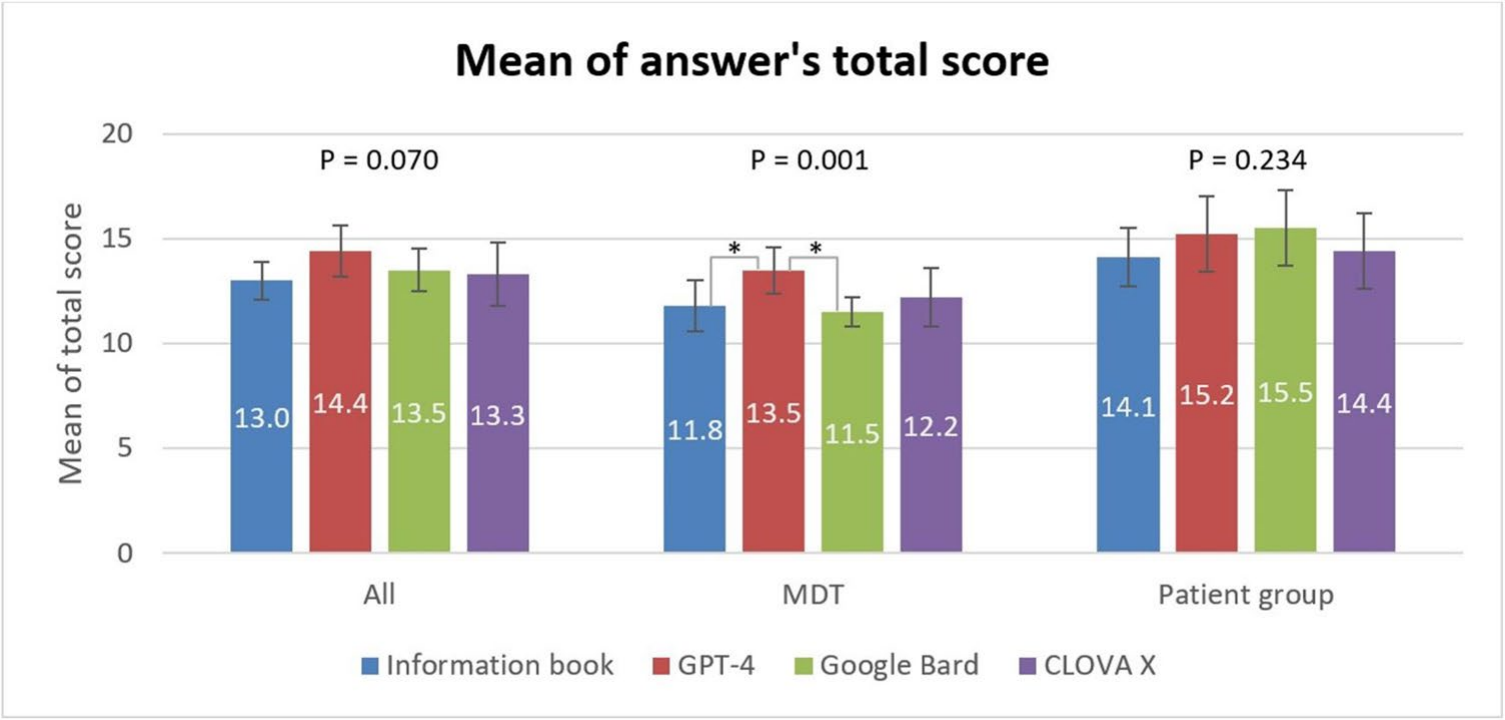
Mean of the total scores for each answer from the responding entities. Among the multidisciplinary team (MDT) evaluators, GPT-4 scores significantly higher (13.5 ± 1.1) than the colorectal cancer (CRC) information book (11.8 ± 1.2, *P* = 0.020) and Google Bard (11.5 ± 0.7, *P* = 0.001) does. Each of the two responding entities significantly different in the post hoc analysis is marked with an *, and the *P*-value of the post hoc analysis between the two responding entities is described in the figure legend. The standard deviation (SD) of the score is shown as an error bar

A comparison of the mean of answer’s total score is presented in Fig. 2. For evaluations that included all evaluators and those limited to the patient evaluators, homogeneity of variance was confirmed, indicating the need for ANOVA; no significant differences were found. However, when only MDT evaluators were included, the data did not meet the assumption of equal variance, necessitating Welch’s *t*-test. The resulting *P*-value was < 0.05, leading to implementation of Games–Howell’s test. Among all evaluators, scores of the CRC information book, GPT-4, Google Bard, and CLOVA X were 13.0 ± 0.9, 14.4 ± 1.2, 13.5 ± 1.0, and 13.3 ± 1.5, respectively, with no significant differences (*P* = 0.070). In the analysis involving only MDT evaluators, GPT-4 scored significantly higher (13.5 ± 1.1) than the CRC information book (11.8 ± 1.2, *P* = 0.020) and Google Bard (11.5 ± 0.7, *P* = 0.001) did, whereas CLOVA X scored 12.2 ± 1.4. In the analysis involving only patient evaluators, the scores of the CRC information book, GPT-4, Google Bard, and CLOVA X were 14.1 ± 1.4, 15.2 ± 1.8, 15.5 ± 1.8, and 14.4 ± 1.8, respectively, with no significant differences (*P* = 0.234).

### Evaluation criteria-specific analysis

The mean scores of the four responding entities were compared among the five evaluation criteria. For accuracy of information scored by only MDT evaluators, the CRC information book, GPT-4, Google Bard, and CLOVA X scored 3.1 ± 0.5, 3.4 ± 0.4, 2.9 ± 0.4, and 3.0 ± 0.6, respectively. No significant differences were observed in the scores obtained for each entity (*P* = 0.064, ANOVA). Regarding “patient satisfaction” scored exclusively by patient evaluators, the CRC information book, GPT-4, Google Bard, and CLOVA X scored 3.9 ± 0.5, 3.9 ± 0.5, 3.9 ± 0.5, and 3.8 ± 0.5, respectively. No significant differences were observed in the scores obtained for each entity (*P* = 0.855, ANOVA).

**Fig. 3:**
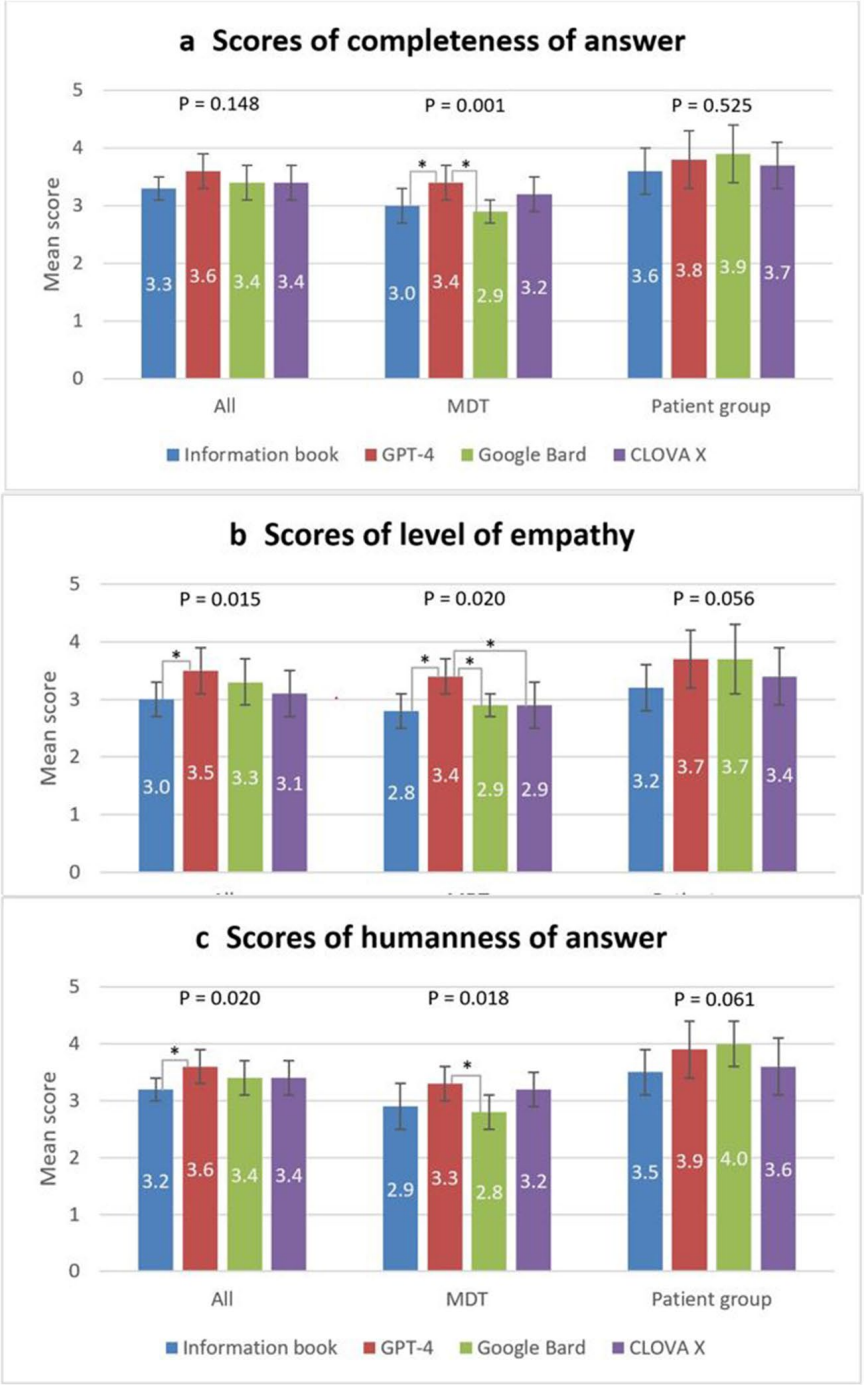
Mean scores of the three evaluation criteria evaluated by the multidisciplinary team (MDT) and the patient group. **a** Completeness of answer: GPT-4 scores significantly higher than Google Bard (*P* = 0.001) and the CRC information book (*P* = 0.009) does, among MDT evaluators. **b** Level of empathy: GPT-4 scores significantly higher than the CRC information book does (*P* = 0.013), among all evaluators. GPT-4 scores significantly higher than the CRC information book (*P* = 0.002), Google Bard (*P* = 0.022), and CLOVA X (*P* = 0.011) does, among MDT evaluators. **c** Humanness of the answer: GPT-4 scores significantly higher than the CRC information book does (*P* = 0.010), among all evaluators. GPT-4 scores significantly higher than Google Bard does (*P* = 0.031), among MDT evaluators. Each of the two responding entities significantly different in the post hoc analysis is marked with an *, and the *P*-value of the post hoc analysis between the two responding entities is indicated above. The standard deviation (SD) of the score is shown as an error bar

The following three criteria were evaluated by the MDT and patient evaluators (Fig. 3). For “completeness of response,” the CRC information book, GPT-4, Google Bard, and CLOVA X scored 3.3 ± 0.2, 3.6 ± 0.3, 3.4 ± 0.3, and 3.4 ± 0.3, respectively, among all evaluators, with no significant differences between the groups (*P* = 0.148). Among MDT evaluators, the CRC information book, GPT-4, Google Bard, and CLOVA X scored 3.0 ± 0.3, 3.4 ± 0.3, 2.9 ± 0.2, and 3.2 ± 0.3, respectively. GPT-4 significantly outperformed the CRC information book (*P* = 0.009) and Google Bard (*P* = 0.001), whereas no significant differences in scores were observed between the other groups. Among patient evaluators, the CRC information book, GPT-4, Google Bard, and CLOVA X scored 3.6 ± 0.4, 3.8 ± 0.5, 3.9 ± 0.5, and 3.7 ± 0.4, respectively, with no significant differences between the groups (*P* = 0.525).

Regarding “level of empathy,” the CRC information book, GPT-4, Google Bard, and CLOVA X scored 3.0 ± 0.3, 3.5 ± 0.4, 3.3 ± 0.4, and 3.1 ± 0.4, respectively, for all evaluators. GPT-4 scored significantly higher than the CRC information book did (*P* = 0.013), whereas no significant differences in scores were observed for the other groups. Among MDT evaluators, the CRC information book, GPT-4, Google Bard, and CLOVA X scored 2.8 ± 0.3, 3.4 ± 0.3, 2.9 ± 0.2, and 2.9 ± 0.4, respectively. GPT-4 scored significantly higher than the CRC information book (*P* = 0.002), Google Bard (*P* = 0.022), and CLOVA X (*P* = 0.011) did, whereas no significant differences in scores were observed between the other groups. Among patient evaluators, the CRC information book, GPT-4, Google Bard, and CLOVA X scored 3.2 ± 0.4, 3.7 ± 0.5, 3.7 ± 0.6, and 3.4 ± 0.5, respectively, with no significant differences between the groups (*P* = 0.056).

Regarding “humanness of response,” the CRC information book, GPT-4, Google Bard, and CLOVA X scored 3.2 ± 0.2, 3.6 ± 0.3, 3.4 ± 0.3, and 3.4 ± 0.3, respectively, among all the evaluators. GPT-4 scored significantly higher than the CRC information book (*P* = 0.010) did, whereas no significant differences in scores were observed between the other groups. Among MDT evaluators, the CRC information book, GPT-4, Google Bard, and CLOVA X scored 2.9 ± 0.4, 3.3 ± 0.3, 2.8 ± 0.3, and 3.2 ± 0.3, respectively. GPT-4 outperformed Google Bard (*P* = 0.031), whereas no significant differences in scores were observed between the other groups. Among patient evaluators, the CRC information book, GPT-4, Google Bard, and CLOVA X scored 3.5 ± 0.4, 3.9 ± 0.5, 4.0 ± 0.4, and 3.6 ± 0.5, respectively, with no significant differences between the groups (*P* = 0.061).

### Question source-specific analysis

**Fig. 4:**
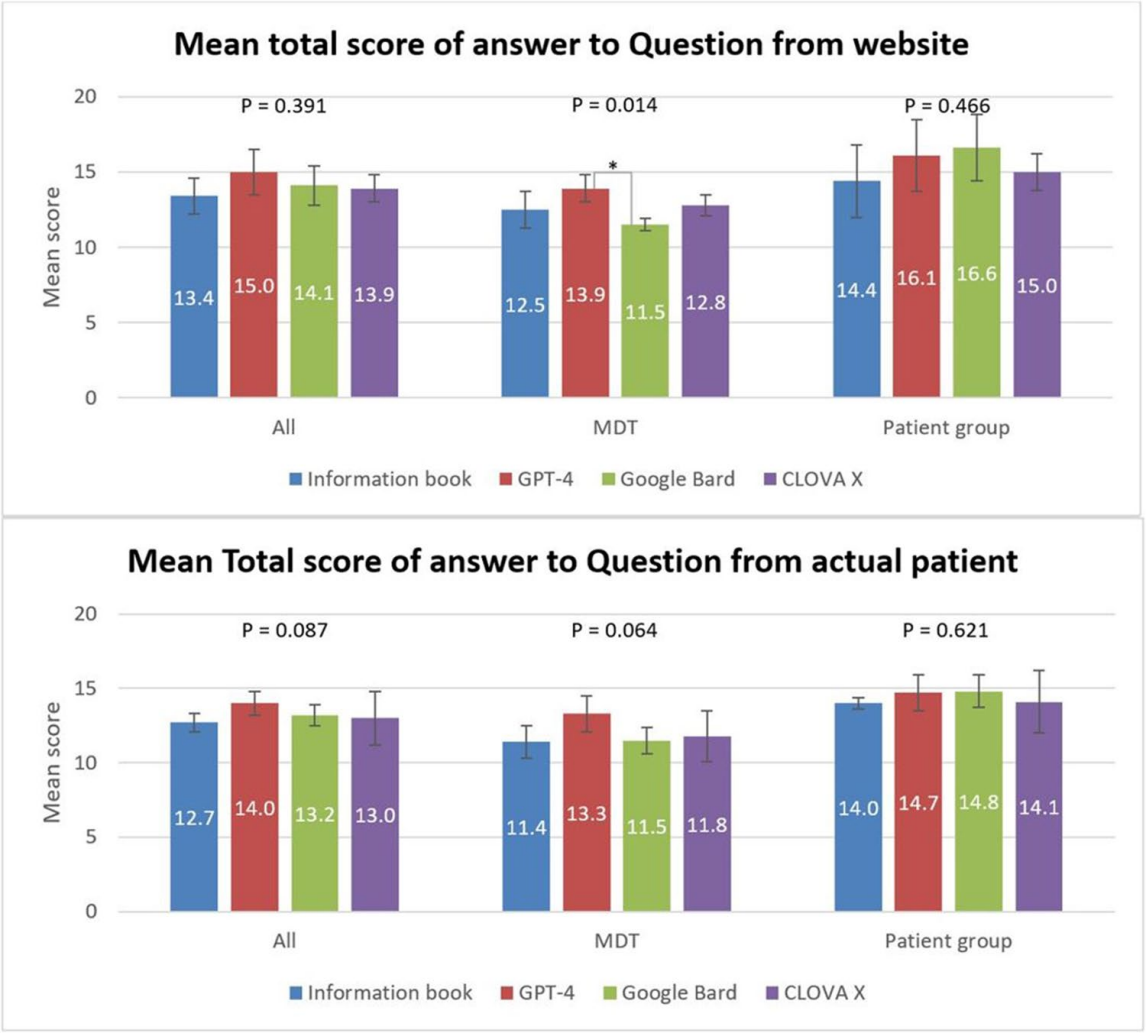
Mean score of answers according to the source of the question. **a** Question from the hospital websites: GPT-4 scores significantly higher than Google Bard does (*P* = 0.008). **b** Question from the patient survey: the analysis confirms no significant differences among all evaluation scenarios, as well as when evaluated separately by either the MDT or patient evaluators. Each of the two responding entities significantly different in the post hoc analysis is marked with an *, and the *P*-value of post hoc analysis between two responding entities is described in this figure legend. The standard deviation (SD) of the score is shown as an error bar

The mean scores obtained by the four responding entities for each group of questions (questions 1–4/questions 5–10) are presented in Fig. 4. For questions sourced from hospital websites (questions 1–4), among all evaluators, the CRC information book, GPT-4, Google Bard, and CLOVA X scored 13.4 ± 1.2, 15.0 ± 1.5, 14.1 ± 1.3, and 13.9 ± 0.9, respectively, with no significant differences between the groups (*P* = 0.391). Among MDT evaluators, the CRC information book, GPT-4, Google Bard, and CLOVA X scored 12.5 ± 1.2, 13.9 ± 0.9, 11.5 ± 0.4, and 12.8 ± 0.7, respectively. GPT-4 outperformed Google Bard (*P* = 0.008), whereas no significant differences in scores were observed between the other groups. Among patient evaluators, the CRC information book, GPT-4, Google Bard, and CLOVA X scored 14.4 ± 2.4, 16.1 ± 2.4, 16.6 ± 2.2, and 15.0 1.2, respectively, with no significant differences between the groups (*P* = 0.466). For questions sourced from the patient survey (questions 5–10), among all evaluators, the CRC information book, GPT-4, Google Bard, and CLOVA X scored 12.7 ± 0.6, 14.0 ± 0.8, 13.2 ± 0.7, and 13.0 ± 1.8, respectively, with no significant differences between the groups (*P* = 0.087). Among MDT evaluators, the CRC information book, GPT-4, Google Bard, and CLOVA X scored 11.4 ± 1.1, 13.3 ± 1.2, 11.5 ± 0.9, and 11.8 ± 1.7, respectively, with no significant differences between the groups (*P* = 0.064). Among patient evaluators, the CRC information book, GPT-4, Google Bard, and CLOVA X scored 14.0 ± 0.4, 14.7 ± 1.2, 14.8 ± 1.1, and 14.1 ± 2.1, respectively, with no significant differences between the groups (*P* = 0.621).

## Discussion

To the best of our knowledge, this is the first study to evaluate the response capabilities of GAI tools to actual patient queries regarding CRC surgery. Our study diverges from previous research, which relied primarily on simulated scenarios, by focusing on real-world patient-centered inquiries. Furthermore, the GAIs’ capabilities were evaluated using a language appropriate for the relevant culture, in addition to English, adding considerable value to the study’s uniqueness.

GPT-4, using the transformer model [17], is adept at tracking relationships within sequential data, e.g., words in sentences, and demonstrates continuous learning and adaptation by integrating human feedback into its evolving responses [9]. When the GAI tool encounters topics beyond its adequate training or supervision scope, it is likely to produce fabricated output, a phenomenon commonly termed as “hallucination [18].” Although hallucination in GAI has not been completely resolved, it has been improved markedly in GPT-4, with some studies reporting a frequency of hallucinations as low as 3% [19, 20]. Google Bard, paralleling the architecture of GPT-4, was constructed using Google’s proprietary LaMDA AI language model. Unlike GPT-4, this model can update information in real-time through Google Search, providing access to the most recent information [21]. However, acceptance of unverified recent information can result in lower accuracy and pose a higher risk of hallucination compared to GPT-4. HyperCLOVA X is a large-scale language model developed by Naver that learns from existing data and further evolves through reinforcement learning based on feedback during conversations [22]. The GAI tool created based on this model is CLOVA X. Given its foundation on a Korean search engine, it has a substantially high proportion of Korean training data. Consequently, it lacks sufficient English data, leading to suboptimal performance in Korean-English translation [23].

ChatGPT (GPT-4) has demonstrated considerable medical knowledge when tested in the context of medical certification examination questions [24, 25]. Particularly, it can respond to medical inquiries of patients in clinical scenarios and is at par with doctors, demonstrating accurate medical knowledge and the ability to interact as a human [7, 16]. Although GAI generally performs well in responding to medical inquiries made by patients, its reliability can vary according to the specific medical subfield [9, 26]. Furthermore, the language being limited to English is a constraint.

**Fig. 5:**
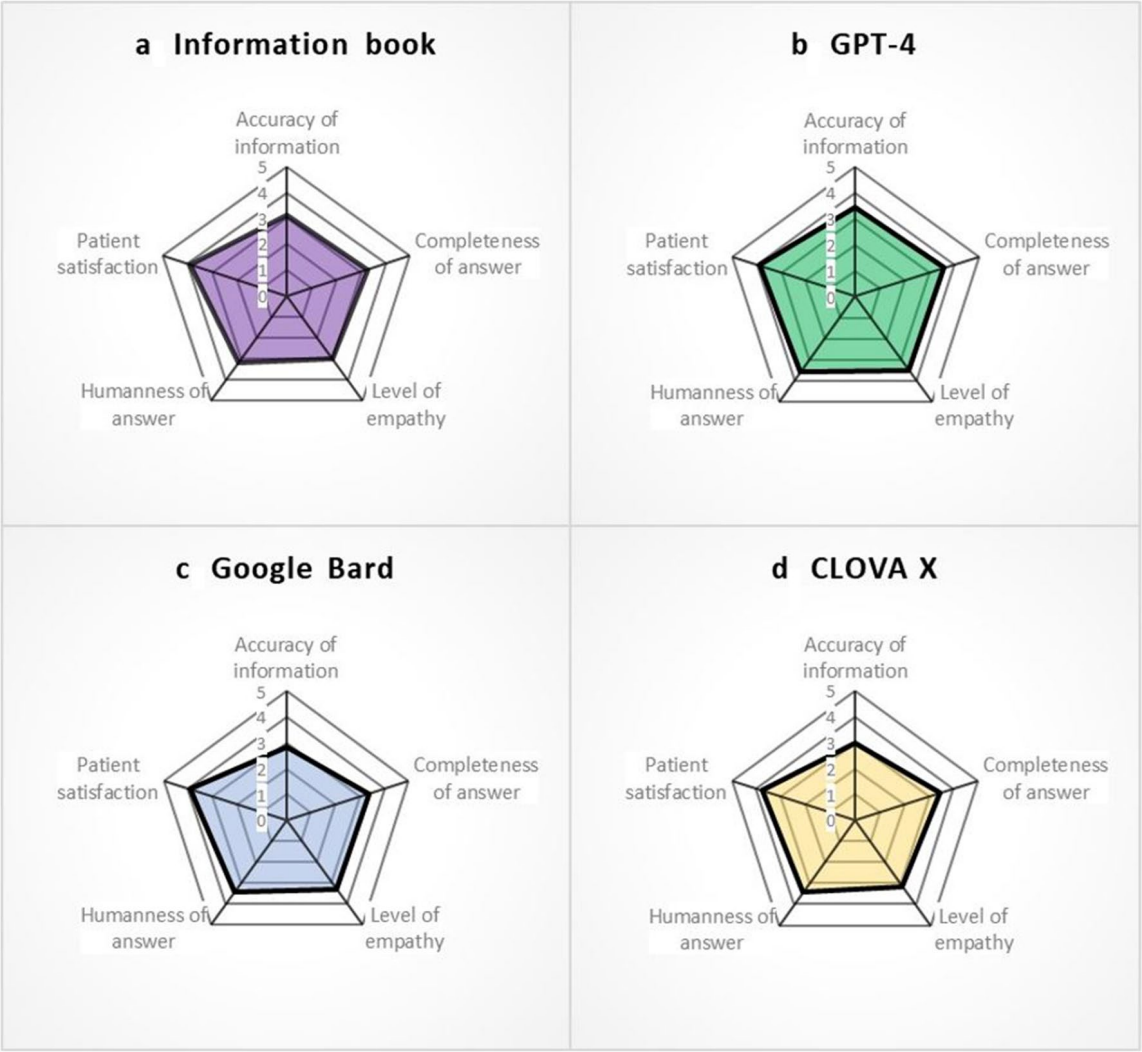
Average score per evaluation criterion for each responding entity. Scores are presented in a clockwise order starting from the accuracy of information. **a** The CRC information book is evaluated with scores of 3.12, 3.3, 2.98, 3.18, and 3.9 in the respective categories; **b** GPT-4 is evaluated with scores of 3.44, 3.61, 3.51, 3.58, and 3.9 in the respective categories; **c** Google Bard is evaluated with scores of 2.86, 3.38, 3.33, 3.41, and 3.94 in the respective categories; **d** CLOVA X is evaluated with scores of 3, 3.42, 3.13, 3.4, and 3.76 in the respective categories. GPT-4 has been verified as occupying the broadest area on the chart, receiving uniformly high scores across the various fields

This study aimed to ascertain whether the competence of GAI in handling questions in English could be replicated for questions in another language, in this case Korean. The radar chart in Fig. 5 displays the scores of the five evaluation criteria for the three GAI tools and the CRC information book. In our study, GPT-4 demonstrated high communication skills across all evaluation criteria without any deficiencies and scored the highest on the total score and on three subscales (completeness of answer, level of empathy, and humanness of answer) assessed by all evaluators and the MDT. Concerning the accuracy of the information, the most important factor in the medical field, GPT-4 scored the highest but did not reach statistical significance when compared with the other GAI tools, which might be a limitation due to the small number of evaluators. This indicates that GPT-4 has medical knowledge that is credible enough for HCPs and a comprehensive understanding from the patients’ perspective. Google Bard displayed commendable performance without a significant difference from that using the CRC information book. Google Bard scored the highest on the total score and on three aforementioned subscales (completeness of answer, level of empathy, and humanness of answer) rated by the patients, although statistical significance was not shown. This suggests that Google Bard has a strong, patient-friendly focus. CLOVA X also demonstrated performance comparable to other GAI tools, which did not show significant differences from the information book. Although CLOVA X is a relatively new developed platform, it is remarkable that it showed capabilities not far behind the more established pioneers. However, as a Korean-based AI, we expected a higher degree of Korean communicative competence from CLOVA X than from other English-based GAI tools; however, this was not the case. Our findings indicate that all the GAI tools evaluated demonstrated the ability to respond at a level similar to that using the CRC information book. The leading performance of GPT-4 is attributed to its pioneering status in the field of GAI, extensive updates, and error corrections.

Prior research has addressed general queries of the population [26–29], often overlooking the specific concerns of inpatients. Our investigation bridges this gap by incorporating direct inquiries from hospitalized patients and questions obtained from online platforms. As presented in Fig. 5, the communicative proficiency of GAI tools aligns closely with that of the established CRC information book, regardless of the source of the questions. The fact that all GAI responses to the website questions were on par with the CRC information book indicates that GAI has effectively learned from extensive data sourced primarily from the Internet in the field of CRC surgery. Interestingly, the ability of GAI to provide answers at a level similar to that of the CRC information book for actual inpatient inquiries, from which they had no prior exposure to learning, suggests an understanding and use of extensive medical data in the field of colorectal surgery. Despite research being conducted in the Korean language, GAI demonstrated impressive response capabilities. This highlights the potential of GAI in facilitating communication between patients and doctors in the Korean healthcare system. Although previous studies have directly evaluated hallucination in GAI [26], our study did not directly assess this aspect. Instead, we indirectly measured the degree of hallucination through the accuracy of the information metric evaluated by the MDT. Efforts will be needed to detail prompt engineering and make questions more intuitive and clearer to reduce hallucinations and obtain more accurate information.

Our study has some limitations. First, the CRC information book used as the baseline for comparison was published in 2020, and most of the information may have been updated in the interim. This factor may potentially lead to an overestimation of the accuracy of GAI and may mistakenly equate its responses with the expert level. Second, the inherent literary style of the CRC information book, as opposed to the conversational tone of GAI, may have influenced the evaluation criteria for the “humanness of answer” and “level of empathy,” potentially biasing the results in favor of GAI. Third, the limited sample size of the study, which included only 10 evaluators and became smaller when divided into MDT and patient groups, may raise concerns about the representativeness of the findings. This small cohort may unintentionally introduce biases on the basis of personal perspectives.

In conclusion, GPT-4, Google Bard, and CLOVA X achieved a level of communicative competence in the Korean language comparable to that achieved using the established CRC information book. However, it is important to consider that patients and their guardians may have limited ability to recognize inaccuracies or hallucinations in the responses provided by GAI. Differences in the GAI responses could significantly impact clinical decisions and long-term patient satisfaction; for instance, inaccuracies in the GAI responses may lead to misinformed patient choices. This could pose risks if these platforms are used autonomously for information-seeking purposes. Therefore, HCPs should strive to create and supervise accurate medical information using GAI. Through these efforts, comprehensive, accurate, and patient-friendly information resources can be created, which would assist patients in accessing reliable medical information and empower them to make informed healthcare decisions.

## Data Availability

No datasets were generated or analysed during the current study.
